# Hyperspectral phenotyping on the microscopic scale: towards automated characterization of plant-pathogen interactions

**DOI:** 10.1186/s13007-015-0073-7

**Published:** 2015-04-15

**Authors:** Matheus Kuska, Mirwaes Wahabzada, Marlene Leucker, Heinz-Wilhelm Dehne, Kristian Kersting, Erich-Christian Oerke, Ulrike Steiner, Anne-Katrin Mahlein

**Affiliations:** Institute for Crop Science and Resource Conservation (INRES) - Phytomedicine, University of Bonn, Meckenheimer Allee 166a, 53115 Bonn, Germany; Department of Computer Science, TU Dortmund, Otto-Hahn-Str. 14, 44227 Dortmund, Germany

**Keywords:** Hyperspectral imaging, Hyperspectral microscope, Spectral reflectance, Plant disease, Disease resistance, Mildew locus o (*Mlo*), Near-isogenic lines, *Hordeum vulgare*, *Blumeria graminis* f.sp. *hordei*

## Abstract

**Background:**

The detection and characterization of resistance reactions of crop plants against fungal pathogens are essential to select resistant genotypes. In breeding practice phenotyping of plant genotypes is realized by time consuming and expensive visual rating. In this context hyperspectral imaging (HSI) is a promising non-invasive sensor technique in order to accelerate and to automate classical phenotyping methods.

A hyperspectral microscope was established to determine spectral changes on the leaf and cellular level of barley (*Hordeum vulgare*) during resistance reactions against powdery mildew (*Blumeria graminis* f.sp. *hordei,* isolate K1). Experiments were conducted with near isogenic barley lines of cv. Ingrid, including the susceptible wild type (WT), mildew locus a 12 (*Mla12* based resistance) and the resistant mildew locus o 3 (*mlo3* based resistance), respectively. The reflection of inoculated and non-inoculated leaves was recorded daily with a hyperspectral linescanner in the visual (400 – 700 nm) and near infrared (700 – 1000 nm) range 3 to 14 days after inoculation.

**Results:**

Data analysis showed no significant differences in spectral signatures between non-inoculated genotypes. Barley leaves of the near-isogenic genotypes, inoculated with *B. graminis* f.sp. *hordei* differed in the spectral reflectance over time, respectively. The susceptible genotypes (WT, *Mla12*) showed an increase in reflectance in the visible range according to symptom development. However, the spectral signature of the resistant *mlo*-genotype did not show significant changes over the experimental period. In addition, a recent data driven approach for automated discovery of disease specific signatures, which is based on a new representation of the data using Simplex Volume Maximization (SiVM) was applied. The automated approach - evaluated in only a fraction of time revealed results similar to the time and labor intensive manually assessed hyperspectral signatures. The new representation determined by SiVM was also used to generate intuitive and easy to interpretable summaries, e.g. fingerprints or traces of hyperspectral dynamics of the different genotypes.

**Conclusion:**

With this HSI based and data driven phenotyping approach an evaluation of host-pathogen interactions over time and a discrimination of barley genotypes differing in susceptibility to powdery mildew is possible.

## Introduction

In agricultural production the demands on efficient crop plants are manifold. Improved quantitative and qualitative plant traits are desired, along with enhanced stress resistance, especially against plant pathogens. The development of resistant cultivars is a challenging task in plant breeding. Fungal plant pathogens affect almost all relevant crops in different stages of their development and impair the yield and product quality. In barley production powdery mildew is one of the main damaging diseases in Europe and other temperate regions [1]. The disease is caused by the biotroph ascomycete *Blumeria graminis* f.sp. *hordei* (*Bgh*) and is spread during the vegetation period by wind with conidiospores. *Bgh* is able to colonize barley plants within 24 hours after the first contact. A haustorium - the feeding organ of *Bgh* – develops within penetrated epidermal cells, which remain vital. The new epiphytic mycelium grows over the leaf surface to penetrate other epidermal cells and develop new haustoria. Finally, *Bgh* produces conidiophores bearing new conidia. This asexual life cycle is completed in approximately five days and is repeated by multiple generations per season. The application of fungicides and the cultivation of resistant barley varieties are the main methods for controlling barley powdery mildew [2]. Unfortunately, the farmers face fungicide resistances developed by *Bgh* [3]. This emphasises the importance of resistant barley genotypes generated in breeding programs.

A well-known resistance mechanism of barley against *Bgh* is the non-race specific mildew locus o (*mlo*) based resistance [4]. In all *mlo* mutants, *Bgh* cannot penetrate the epidermal cell, because a cell wall apposition (papilla) is developed under the penetration point [5] that possesses a high electron density [6]. The basic components of this cell wall apposition are a complex of lignin, cellulose, callose, peroxidases, phenols, proteins and further cell-wall materials [7]. The mildew locus a (*Mla*) gene based resistance is an another resistance reaction of barley against *Bgh* and is associated with a hypersensitive reaction of epidermal cells attacked by *Bgh* [7-9].

These resistance properties are used in plant breeding programs to improve the resistance of barley plants to powdery mildew. However, one main drawback of recent breeding programs is their time consuming and labor intensive nature. The traditional breeding procedure of common crop plants still takes 7 to 17 years and requires a high amount of plant material and human effort on the way to a desired cultivar [10]. In this complex breeding process, manifold steps in different environments - under controlled and under field conditions - at different plant levels - from single organs to the canopy - are executed. Hereby the selection process of predominant genotypes and relevant crop traits by genotyping and phenotyping methods is crucial and determines the time span and the success of the breeding process.

In recent years the genotyping of plants has been significantly accelerated by advances in molecular profiling and sequencing technologies [11]. Marker-assisted selection, bi-parental recombinant inbred lines, or the increasing number of completely sequenced species in genomic databases provides solutions to current breeding challenges [12]. To bridge the gap from genomic characterization to plant function and agricultural traits, the expression of the genome in a given environment has to be tested carefully. This step is defined as the phenotyping process. Several authors have addressed the labor-intensive and costly nature of conventional phenotyping processes as the limiting and time-consuming factor in plant breeding. This challenge has been identified as the phenotyping bottleneck [11-13].

Innovative technologies, e.g. optical and non-invasive sensors, have been characterised as new phenotyping methods with potential to overcome this bottleneck and to improve the breeding process. Various optical imaging methods using e.g. RGB [11], 3D [14], fluorescence [15], thermography [16] and HSI [17] sensors are able to characterize different plant parameters and could potentially be implemented in automated, high-throughput phenotyping pipelines.

Among these methods, hyperspectral imaging (HSI) is one of the most promising techniques to assess functional plant traits [17-19]. Using HSI, the spectral characteristics of plants can be visualised non-invasively over time and on different scales. The sensitivity of sensors enable a high spectral and spatial resolution and the reflectance per pixel can be analysed in narrow wavelengths. Characteristic spectral signatures provide information about the physiological status of plants and for plant breeding on the reaction of different genotypes to biotic or abiotic stress factors [20]. Leaf pigments, like chlorophylls and carotenoids are the main factors influencing the spectral information in the visual range (VIS, 400–700 nm) [21]. The near infrared range (NIR, 700–1000 nm) is mainly affected by scattering processes in the spongy mesophyll, and additionally by absorptions due to proteins, fatty acids, starch, water and cellulose with different specific absorption wavelengths. The leaf water content, including further chemical compounds, can be derived from the short wave infrared range (SWIR, 1000–2500 nm).

Various researchers have demonstrated that plant diseases and processes during pathogenesis can be detected by hyperspectral sensors [22-24]. Plants diseased by fungal pathogens could be distinguished from healthy plants at different stages of the pathogenesis and at different disease severities. Since first interaction sites and primary symptoms of fungal plant diseases are in the range of sub-millimeters, highly sensitive sensor systems and powerful subsequent data analysis routines are required for a reliable evaluation of plants under biotic stress. Few researchers postulate, that early modifications of the cellular leaf structure occur due to fungal toxins or plant resistance reactions and that these subtle changes are detectable via HSI [25,26]. They focused on small-scale studies with HSI or on the detection and identification of plant diseases before visible symptoms appear. Based on these insights the development of a HSI routine to estimate the susceptibility of plants and to characterise defence mechanisms against fungal pathogens is a desirable task. However, it requires methodological adaptions and technical advances to exploit the potential of hyperspectral sensors for the implementation in resistance screenings.

The hypothesis of this study is that subtle processes during compatible and incompatible plant-pathogen interactions have an effect on optical properties of plants. It is expected that specific regions of the electromagnetic spectrum are influenced depending on the type of interaction. The detection of these changes demands specific sensor setups with a high spatial and spectral resolution combined with sophisticated, data analysis methods. To proof this hypothesis a HSI microscope, a measuring protocol for detached barley leaves and an automated data analysis approach was established in this study. This approach can be used in resistance screening for the differentiation of barley genotypes and for a characterization of their interaction with *Bgh*, the causal agent of powdery mildew. As an application model susceptible and resistant genotypes with different, well known genetic backgrounds were monitored visually and by hyperspectral imaging in time-series experiments at small-scale level.

To uncover the full information from high-dimensional HSI data, characteristic spectra were extracted both manually and using data mining techniques. A qualitative and automated analysis of reflectance data was realized using a data driven approach based on the matrix factorisation technique Simplex Volume Maximisation (SiVM) [27]. With this methodological approach, (i) processes during pathogenesis on the different genotypes could be characterized, (ii) stages of pathogenesis were automatically visualized and (iii) spectral dynamics were evaluated over time.

## Results and discussion

**Figure 1:**
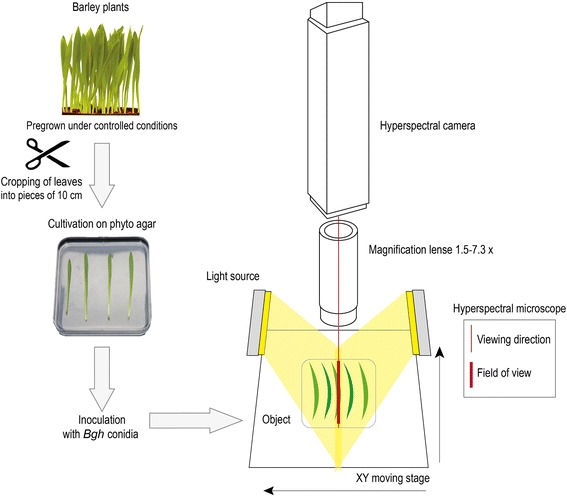
Hyperspectral imaging microscope setup for small-scale image analysis. The spectral reflectance of detached barley leaves on phyto agar was measured with a hyperspectral camera, which was mounted on a magnification lens to enable a magnification up to 7.3x. Two linear light emitters with a vertical orientation of 30° illuminated the samples in a distance of 20 cm. The samples were moveable due to a XY moving stage. A spectral resolution of up to 2.73 nm and a maximum spatial resolution of 7.5 μm per pixel was obtained. The field of view ranged from 4.1 to 0.95 cm, dependent on the magnification.

The pathogenesis of powdery mildew and early interactions on different barley genotypes was characterized on the leaf and tissue level using a hyperspectral microscope (Figure 1). A high spatial resolution of a pixel size as low as 7.5 μm allowed the detection of subtle processes.

Hyperspectral imaging can improve disease detection through a better examination of host pathogen interactions [28]. This has been demonstrated by researchers, using a diversity of hyperspectral sensors with different crops and their relevant diseases on different scales ranging from remote to proximal sensing [24,29]. Since imaging sensors allow for a pixel-wise attribution of disease-specific symptoms, primary infection sites can be identified and analyzed spectrally [25]. In contrast to our new plant phenotyping approach, existing HSI microscopes are prohibited due to destructive nature [30,31].

### Phenotypic development of healthy and inoculated barley leaves

**Figure 2:**
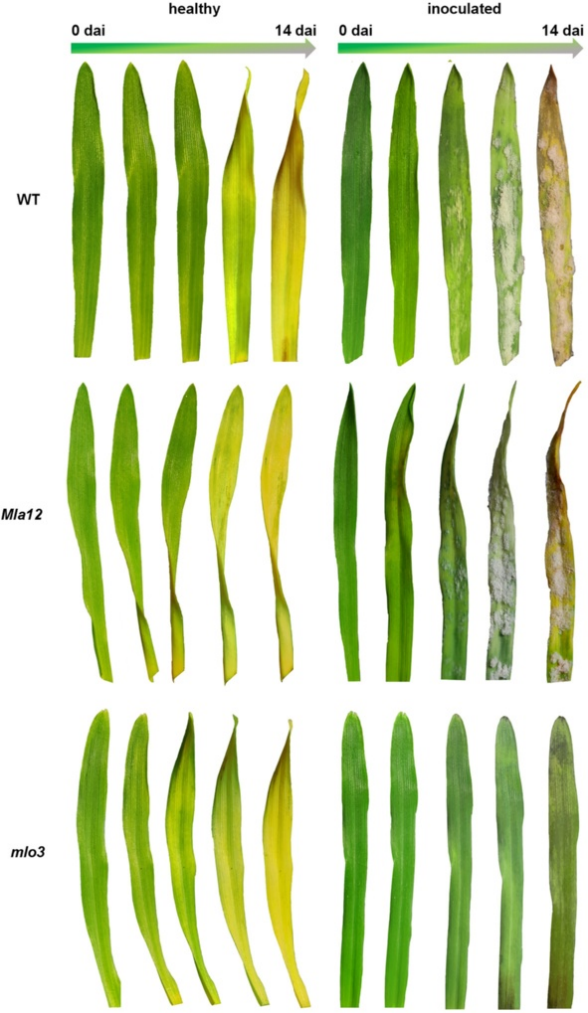
Phenotypes of detached barley leaves non-inoculated (healthy) and inoculated with *Blumeria graminis* f.sp. *hordei* (*Bgh*) of near-isogenic lines cv. Ingrid (WT, *Mla12* and *mlo3*) 0, 3, 6, 10 and 14 dai, on phyto agar. Non-inoculated leaves of the genotypes showed natural senescence over the experimental period. First characteristic powdery mildew pustules became visible 6 dai on inoculated leaves of the susceptible WT and the near-isogenic line *Mla12*. The resistant near-isogenic *mlo3* leaves did not show any powdery mildew symptoms. Senescence of inoculated near-isogenic *mlo3* leaves was delayed until 14 dai. Images were taken with a digital camera (EOS 6D, Canon, Tokio, Japan) and a 100 mm object lens (EF Lens Ultrasonic EF 100 mm 1:2.8 L Macro IS USM, Canon, Tokio, Japan).

The phenotypes of detached, healthy and *Bgh* inoculated leaves of barley genotypes WT, *Mla12* and *mlo3* were assessed visually on phyto agar (Figure 2). *Mla12* leaves were included as an additional susceptible genotype to analyze differences during the pathogenesis between near-isogenic lines, respectively. Non-inoculated leaves of the three genotypes did not show any visible symptoms during the first 6 days after inoculation (dai). Symptoms of senescence occurred 6 dai on healthy *mlo3* leaves, indicated by yellowing of the leaves. The WT and *Mla12* started to became chlorotic 10 dai. This senescence process developed further until 14 dai. The relative long life span and vitality of detached leaves in this investigations indicated consistent conditions for HSI of the plant system in a controlled environment, for a period of 14 days.

*Bgh* inoculated leaves showed no visible symptoms during the first 5 dai. Characteristic powdery mildew pustules occurred 6 dai on the susceptible WT and the near-isogenic *Mla12* line. On *Mla12* leaves the pustules were distributed homogeneously on the leaf surface compared to clustered pustules on WT leaves. Pustules expanded and covered nearly the complete leaf surface of the susceptible WT and *Mla12* leaves 10 dai. Furthermore, the leaves became light-green and chlorotic in areas without powdery mildew pustules 10 dai. Necrotic tissue occurred with the exception of powdery mildew dominated leaf areas, which showed light-green to yellow discoloration 14 dai. The resistant *mlo3* leaves did not show any powdery mildew symptoms during the experiment. The leaves were healthy and green with a delayed senescence. First signs of senescence of inoculated *mlo3* leaves appeared only 14 dai. Further studies are required for an explicit interpretation of the decelerated senescence of the inoculated *mlo3* leaves.

### Spectral similarity of non-inoculated near-isogenic barley lines over time

**Figure 3:**
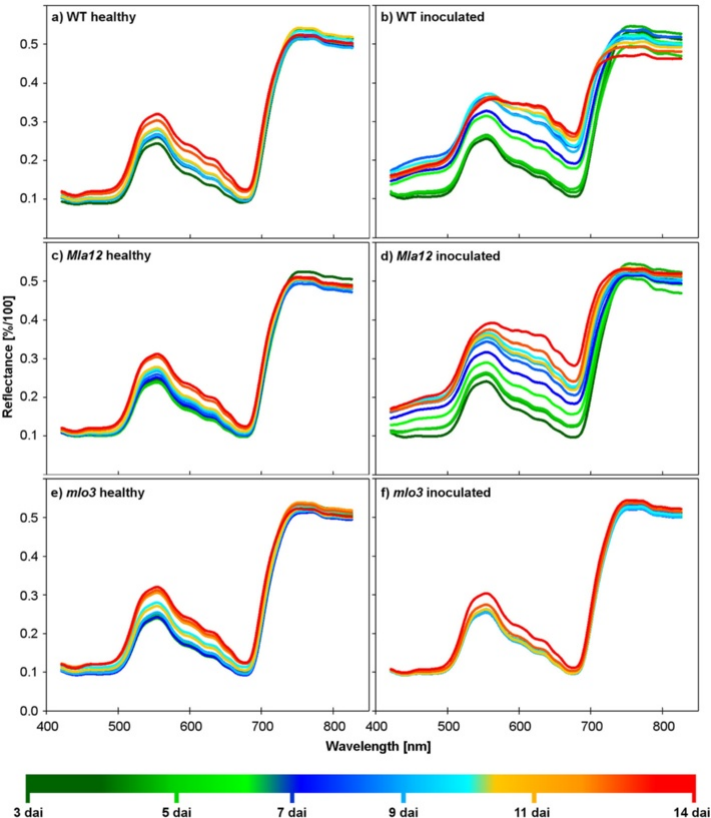
Spectral signatures of non-inoculated (healthy) barley leaves cv. Ingrid WT **(a)**, *Mla12* **(c)**, *mlo3* **(e)** and barley leaves inoculated with *Blumeria graminis* f.sp. *hordei* (*Bgh*) **(b**, **d**, **f)**, from 3 to 14 dai. Reflectance spectra of healthy leaves of the near-isogenic lines are similar. During the measuring period, the reflectance of healthy leaves increased in the visible range. Reflectance of inoculated, susceptible genotypes (WT and *Mla12*) increased in the VIS and WT had decreased reflectance in the NIR. The inoculated, resistant *mlo3*-genotype showed significant differences to healthy leaves in the reflection from 530 – 680 nm only 14 dai. (n = 3).

Detached non-inoculated (healthy) and inoculated leaves of the near-isogenic lines cv. Ingrid WT, *Mla12* and *mlo3* were measured daily 3 to 14 dai in order to assess changes in the spectral signatures. Healthy leaves of the different near-isogenic lines exhibited a typical spectral pattern of healthy plants with low reflectance from 400 – 700 nm, a characteristic green peak at 500 – 570 nm, a steep reflectance increase at the red edge inflection point and a high reflectance plateau in the NIR 3 days after detachment (Figure 3a). This pattern slightly changed over time. The reflectance between 420 and 680 nm increased every day due to changes in the pigment composition [32-34]. Other ranges of the spectrum were not affected (Figure 3a). Spectral changes indicated senescence processes of non-inoculated, healthy leaves over time, which were in accordance to the leaf phenotype. However, differences in the development of individual leaves were reflected by the relative standard deviation (RSD) of 0.6 – 6.8% over time and measured wavelength range. The highest RSD was calculated in the NIR range for WT 10 dai, for *Mla12* 13 dai, and for *mlo3* 13 dai. The low RSD indicate the practicability and robustness of a detached leaf system for the assessment of phenotypic differences due to resistance reactions. The similarity between reflectance spectra of healthy *Mla12* and *mlo3* leaves to healthy WT leaves was due to the identical genomic background of the near-isogenic lines.

Consequently, healthy leaves of the near-isogenic lines cv. Ingrid *WT*, *Mla12* and *mlo3* showed a high spectral similarity and a similar performance on the phyto agar plates. The assessed barley spectra were characteristic reflectance patterns of healthy plant tissue [25,26,35,36]. Reflectance of the detached leaves between 420 – 740 nm increased with each day due to senescence. The absorption features in this range are related to chlorophyll and other pigments linked to photosynthesis [21,33,34]. Increased hyperspectral reflectance indicated a reduction to the chlorophyll activity and content. This effect is well described as one main process during plant senescence [32]. The course of reflectance changes due to senescence coincided with the phenotypic senescence processes observed (Figure 2).

### Spectral signatures of near-isogenic barley lines during powdery mildew pathogenesis

The susceptible near-isogenic genotypes cv. Ingrid WT and *Mla12* and the *Bgh* isolate K1 were used to evaluate the progress of powdery mildew pathogenesis and to identify spectral fingerprints of the barley-*Bgh* system. Inoculated WT leaves showed minor differences to healthy WT leaves 3 dai (Figure 3b). The reflectance of inoculated WT leaves increased between 534 – 563 nm. An overall increase of reflectance in the entire range was observed already 4 dai and the shift to higher reflectance, continued the following days. Within this period of time, the increased reflectance was in accordance with the *Bgh* ontogenesis on barley leaves. The reflectance alterations in the VIS of inoculated WT leaves indicated changes in photochemical processes and pigment content, which are associated to the photosynthetic activity [32-34]. Reflectance in the NIR from 743 – 830 nm decreased 5 dai. This NIR response turned to an increased reflectance again 6 dai, when first powdery mildew pustules occurred on the WT leaf surface. Subsequently, the reflectance in the NIR from 743 – 830 nm decreased stepwise until 14 dai. Symptoms were accompanied by significant reflectance changes over the full range. This gradual increase of reflectance was prominent from 400 – 680 nm and from 700 – 740 nm. The reflectance in the VIS increased daily according to the growth of powdery mildew mycelium until 9 dai. The course of the spectral pattern changed from 10 to 14 dai and the reflectance from 420 – 500 nm decreased again due to the occurrence of first necrosis and tissue collapse below powdery mildew pustules. Increased green reflectance was in accordance with senescence chloroses, associated to a reduction and breakdown of chlorophyll (Figure 2). The reflectance spectrum 14 dai represented a necrotic leaf tissue covered with powdery mildew. The RSD among the inoculated WT leaves was 1 – 14.7% over time and wavelengths. The highest RSD was calculated 14 dai over the full spectral range. The spatial distribution of the *Bgh* mycelium and the vitality of the individual leaves influenced the leaf phenotypes, which explained the higher RSD of the hyperspectral reflectance compared to non-inoculated leaves.

The hyperspectral reflectance pattern of *Bgh* pathogenesis described for WT was also monitored for the near-isogenic line *Mla12* (Figure 3d). The appearance of first tiny powdery mildew pustules 4 dai was associated to first increase of leaf reflectance between 420 to 680 nm. In contrast to inoculated WT leaves, reflectance did not decrease in the blue range 10 dai and later. Reflectance of *Mla12* leaves in the range 500 – 742 nm increased day by day in contrast to the WT leaves. This effect can be explained by a faster development of *Bgh* on *Mla12* leaves compared to WT leaves. In addition, the diseased area and the density of mycelium and conidia on the leaves was higher than on the WT leaves. Similar to inoculated WT leaves, reflectance increased stepwise until 14 dai, except from 743 to 830 nm the reflectance did not change significantly over time. Interestingly, the reflectance between 743 to 830 nm 5 dai was lower compared to the other days. This phenomenon was also observed for inoculated WT leaves 5 dai. The RSD of reflectance among inoculated *Mla12* leaves was 3 – 14% over time and wavelengths. The highest RSD was calculated between 500 – 680 nm 14 dai. Also the range 420 – 500 nm showed high RSD among the inoculated *Mla12* leaves from 6 dai until 14 dai.

In contrast, susceptible near-isogenic lines WT and *Mla12* showed slightly differences in the spectral reflectance during the pathogenesis. Nevertheless, reflectance patterns of *Bgh* pathogenesis on the susceptible genotypes were characterized by a reflectance increase between 400 – 700 nm over time. This increase is due to white powdery epiphytic mycelium and conidia. Similar patterns were observed for powdery mildew diseased leaves of sugar beet and winter wheat on different scales [25,37]. The results demonstrate a similarity of spectral patterns and dynamics during powdery mildew pathogenesis, independently of the scale of investigations, but with a higher sensitivity of the HSI microscope because of the higher spatial resolution.

### *Mlo3* inoculated leaves showed no powdery mildew infestation over time

The spectral reflectance of *Bgh* inoculated *mlo3* leaves (Figure 3f), differed from that of inoculated, susceptible WT and *Mla12* leaves. Resistant *mlo3* leaves showed a spectral pattern similar to non-inoculated leaves of all genotypes until 9 dai. No visible symptoms were assessed on the inoculated *mlo3* leaves. Interestingly, inoculated *mlo3* leaves showed no effects of natural senescence on reflectance until 13 dai. The reflectance was constant over time with low RSD of 5-7% in the full spectral range. A first increase in reflectance was observed from 540 – 680 nm 13 dai, and first symptoms of senescence occurred.

Swarbrick *et al*. [38] reported an induced cell-death and a reduction of the photosynthetic activity during the resistance reaction of *mlo5* leaves inoculated with *Bgh* isolate A6. In contrast, *Bgh* isolate K1 inoculated *mlo3* leaves in this study did not change the chlorophyll content until 13 dai. This is indicated by constant low reflectance from 420 – 680 nm [32-34]. Moreover, the constant reflectance over time, especially at 680 – 700 nm, allowed to distinguish between susceptible and resistant leaves already 4 dai. Specific resistance reactions of barley genotypes, such as lignification, controlled cell death, or formation of papilla may be assessed only by using a HSI system with higher spatial resolution [25]. The hyperspectral microscope is an important methodological innovation to elucidate subtle responses of plants to biotic stress. However, specific reflectance patterns of the barley *mlo3* resistance reaction were not assessed in this experiment. Further investigations of the first 48 hours after inoculation are required, since most defense mechanisms of plants against fungal pathogens take place within the first hours after plant-pathogen-contact, attempted penetration and early infection [39]. The *mlo5* based papilla formation against *Bgh*, for instance is completed within the first 16 – 30 h after the contact of conidia and plant surface [9]. To improve the results from the HSI microscope exact spatial referencing of images from subsequent days is necessary. A better spatial orientation within the image could be realized by placing localization plates beside the leaf area of interest.

### An automated approach for hyperspectral image analysis for plant phenotyping

**Figure 4:**
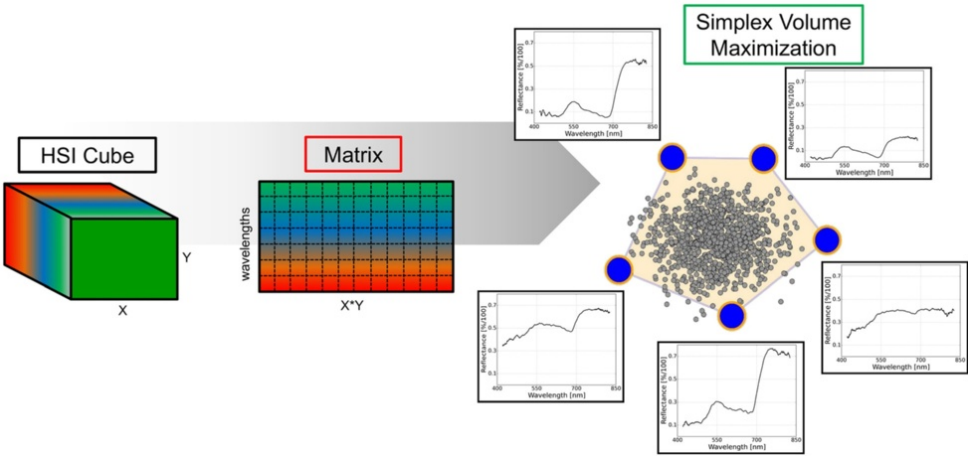
Interpretable matrix factorization for hyperspectral images. Each hyperspectral data cube is transformed into a dense matrix. Then, extreme components/signatures on all matrices are computed, using Simplex Volume Maximization. The final step includes the computation of the new representation of all signatures in a space, spanned by the extremes.

**Figure 5:**
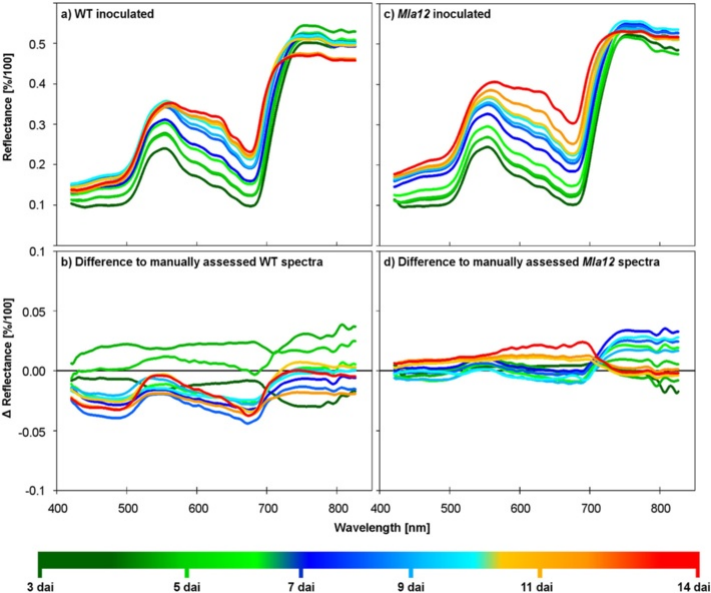
Automatically determined mean signatures of barley leaves cv. Ingrid WT **(a)** and *Mla12* **(c)** inoculated with *Blumeria graminis* f.sp. *hordei* from 3 to 14 dai. The automatically assessed spectra were similar to signatures assessed manually. The differences between automatically and manually analyzed data for WT were −0.04 – 0.04 [%/100] **(b)**, −0.02 – 0.03 [%/100] for *Mla12* **(d)**, respectively, over the wavelengths and time.

The effective analysis and interpretation of HSI data are limiting factors for an implementation into plant phenotyping [36]. The 3-dimensional complexity of HSI data requires high input of human intervention and labelling of disease specific image pixels. Automated analysis pipelines are required to optimize the use of HSI for phenotyping or precision agriculture. Within this study an automated analysis cascade using Simplex Volume Maximization was adopted (Figure 4). Reflectance spectra of inoculated WT leaves assessed by this automatic approach (Figure 5a) showed patterns similar to the manually assessed reflectance spectra. Differences between manually and automatically extracted reflectance were calculated to highlight the similarity of the results (Figure 5b,d). According to both methods of data analysis, no differences between healthy and diseased leaves were observed 3 dai. An increase of reflectance between 420 and 742 nm was detected in the automated approach already 4 dai indicating a higher sensitivity when spectral information is defined automatically from all pixels of an image. Further differences were detected in the NIR range 4 and 5 dai (Figure 5b). The next days showed a spectral trend similar to the manual analysis. The differences between automated and manually assessed reflectance values were low and reached only −0.04 [%/100] at 680 nm. The automatically assessed reflectance spectra of inoculated *Mla12* leaves were in accordance to those assessed manually (Figure 5c); differences varied from −0.02 to 0.03 [%/100] over time and wavelengths (Figure 5d). The present results highlight a standardized system with detached leaves for a HSI microscopy and automated data mining suitable for plant phenotyping. Interestingly, the automatically assessed reflectance spectra showed more details among days (Figure 5) as all image pixels were considered, whereas the manually assessed spectra are only from a few selected pixels.

**Figure 6:**
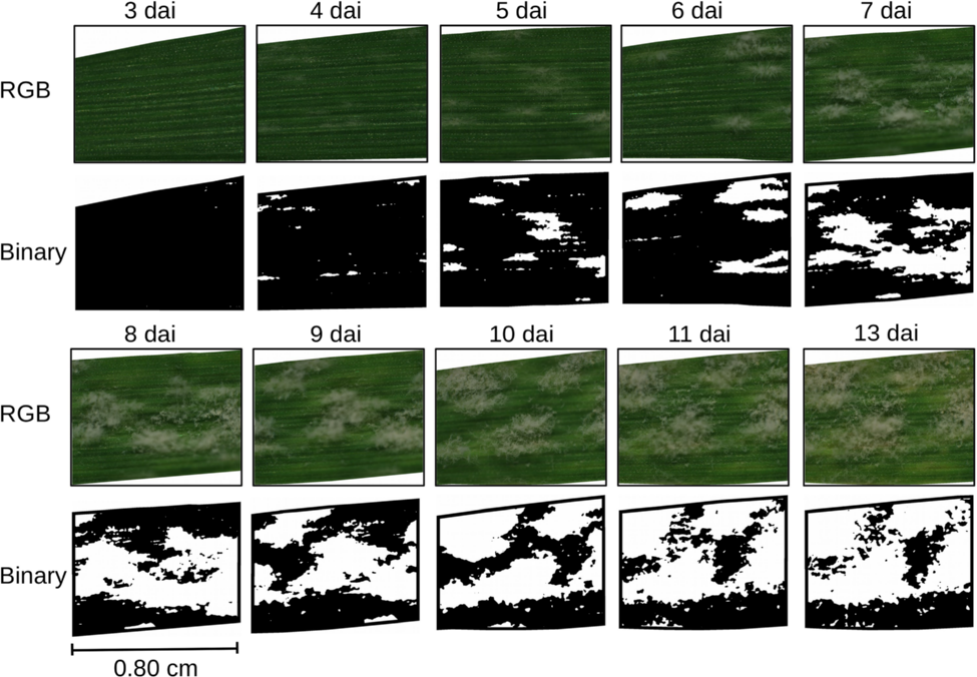
RGB images and binary infestation maps for automatic localization of barley tissue diseased by *Blumeria graminis* f.sp. *hordei* 3 to 13 dai. Black color indicates *Bgh* free tissue, white color highlights *Bgh* diseased barley tissue. Image section varies from day to day. No powdery mildew symptoms were visible 3 dai on RGB images. The binary map was almost completely black with small exceptions. First tiny powdery mildew pustules occurred in the RGB image 4 dai, which were detected and illustrated as white areas on the binary map.

Based on the automatically assessed reflectance spectra a binary map system of *Bgh* inoculated susceptible leaves was established (Figure 6). The binary maps visualize *Bgh* diseased leaf tissue over time. Black pixels indicate healthy leaf tissue, while white pixels indicate sites with powdery mildew. This allows the observation of disease development on susceptible plant genotypes with rapid visual identification of relevant pixels. Powdery mildew symptoms were absent on RGB images 3 dai. The corresponding binary map was almost completely black, however some white pixels appeared before visible symptoms occurred. First tiny powdery mildew pustules became visible on RGB images 4 dai and were accurately detected in the binary map. Senescent leaf tissue was not included in the binary maps due to the consideration of natural senescence of detached, healthy leaves. Characteristic spectral patterns could be identified without human intervention. The binary maps illustrates disease specific pixels and allows the operator to control the automated results by comparing the binary maps with the corresponding RGB images. In complex biological systems and for resistance screenings, it will be an advantage to take spatial properties of spectral dynamics into account [25,40]. This unsupervised and data driven approach requires no a-priori knowledge such as pre-defined endmembers from a spectral libraries used in existing classification or machine learning approaches [20].

### Extraction of spectral dynamics of healthy and *Bgh* inoculated barley leaves

**Figure 7:**
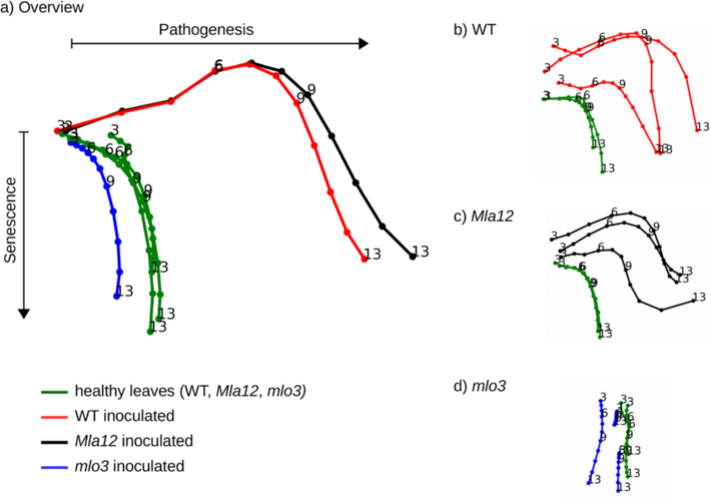
Leaf traces to uncover hyperspectral dynamics of healthy and *Blumeria graminis* f.sp. *hordei* inoculated near-isogenic lines of cv. Ingrid leaves (WT, *Mla12*, *mlo3*) over time. Healthy leaves had a similar spectral pattern and trend, indicated by a minor distance among their traces **(a)**. This is shown also for inoculated WT and *Mla12* leaves **(a)**. Between the individual leaves, hyperspectral dynamics are illustrated by their spectral traces **(b**, **c)**. The traces of inoculated, resistant *mlo3* leaves differed from healthy and inoculated, susceptible (WT and *Mla12*) leaves over the measuring period **(a)**. Inoculated *mlo3* leaves, showed differences **(d)**.

The spectral dynamics of healthy and inoculated near-isogenic lines over time were used to elucidate differences among the near-isogenic lines illustrated as traces (Figure 7) according to Kersting *et al*. [41]. The spectral traces are an example of an interpretable summary of high dimensional hyperspectral imaging data, highlighting the phenotypic evolution and processes during the interaction of *Bgh* with susceptible and resistant barley genotypes. With this interactive approach, an adaption of known data mining methods to plant phenotyping tasks is demonstrated.

Figure 7a illustrates the mean traces of healthy and inoculated WT, *Mla12* and *mlo3* genotypes. Each line describes the spectral trace of three leaves 3 to 13 dai. The similarity among healthy leaves is indicated by the close trends of the traces. They developed in the same direction and had similar dynamics in time, indicated by short traces. Differences in the spectral traces of diseased WT and *Mla12* leaves were apparent. A variation in symptom development and time can be concluded from slightly different traces of the inoculated genotypes. During the experimental time, the mean trace of resistant *mlo3* overlapped with the traces for all healthy leaves and finally differed from them. The traces in Figure 7a allowed for an overall overview of the disease development over time. In order to reach this global view, we used the averaged mean signatures over all images of the leaves of the same type and treatment. To visualize specific details (Figure 7b-d), traces for each particular leave are provided for each genotype (WT, *Mla12* and *mlo3*) and treatment (healthy and inoculated) separately.

Differences in the spectral traces in direction and length, between the healthy and diseased genotypes are indicated (Figure 7b,c). For inoculated, susceptible genotypes WT and *Mla12*, differences within genotype and treatment resulted from different disease severities and development stages of powdery mildew over time. Likewise, inoculated *mlo3* leaves gave different spectral traces (Figure 7d).

## Conclusions

**Figure 8:**
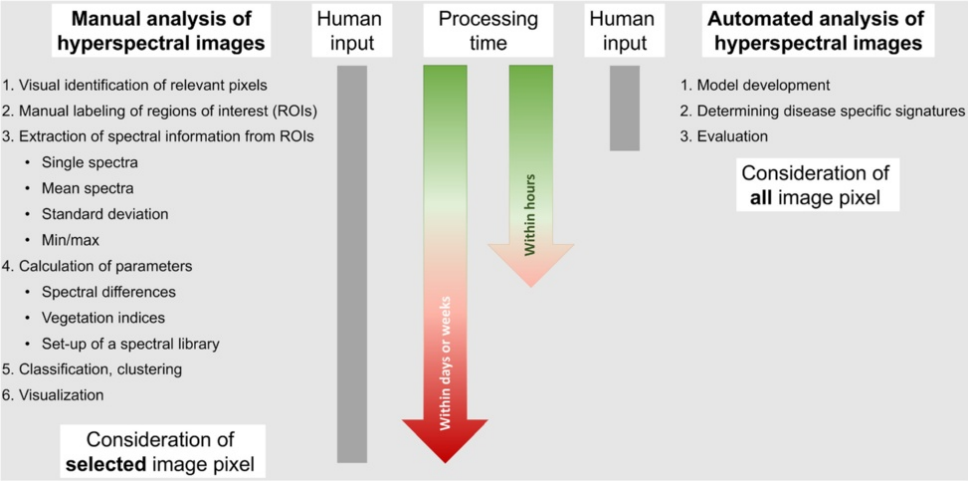
Workflow of the manual and automated hyperspectral image analysis, starting after preprocessing of hyperspectral images. Manual analysis requires high input of human experts and hence is time and cost intensive while still subjective. The automated analysis cascade improves the analysis of hyperspectral images due to the reduction of human input, the economization of time and the consideration of all image pixels.

Spectral information and phenotypes, assessed with a detailed microscopic HSI approach correspond to reflectance data from single plants or crop stands, whereby the HSI microscope enables a higher spatial resolution and richness of details. In microscopic HSI, the small pixel size (7.5 μm) eliminates the problem of pixels with mixed information from initial sites of pathogen infection. The proposed phenotyping set up is a promising new approach for the hyperspectral assessment and characterization of plant diseases and early processes during pathogenesis. In incompatible host-pathogen systems, specific resistance reactions may be identified from spectral reflectance data. The data analysis cascade based on data driven, automated machine learning methods, reduces the required human input in disease resistance screening systems (Figure 8) and in the evaluation of the performance of a set of plant genotypes under different environmental conditions.

## Materials and methods

### Plant cultivation and inoculation of *Blumeria graminis* f.sp. *hordei*

Near-isogenic barley (*Hordeum vulgare*) lines cv. Ingrid-wild type (WT), Ingrid -I10 containing resistant mildew locus a 12 (*Mla12*) [42] and Ingrid -M. C. 20 containing resistant mildew locus o 3 (*mlo3*) [43] were grown in a commercial substrate (Klasmann-Deilmann GmbH, Germany) in plastic pots (10×10×20 cm) in a greenhouse at 23/20°C (day/night), 60% relative humidity (RH) and a photoperiod of 16 h. One week after sowing, the primary leaves (with an approx. length of 10 cm) were detached and transferred on to aseptic phyto agar (Duchefa Biochemie, Haarlem, Netherlands) containing 0.034 mM benzimidazole.

For each genotype, three leaves were inoculated with fresh spores of *Blumeria graminis* f.sp. *hordei* isolate K1 (*Bgh*-K1), and four leaves were kept untreated as healthy control. *Bgh-K1* is virulent to cv. Ingrid WT and Ingrid I10 [42] and avirulent to Ingrid M. C. 20 [43]. Fresh conidia were obtained from heavily infected barley (cv. Leibniz). Twenty-four hours before plant inoculation, the conidia of *Bgh*-K1 infested plants were shaken off and discarded in order to assure homogenous and vital conidia for the inoculation. Conidia of a recently formed powdery mildew pustule (7 dai) are transferred to the prepared leaves on phyto agar using an aseptic inoculation loop. The agar plates were sealed with Parafilm M® (Bemis, Oshkosh, USA) and incubated in a controlled environment at 19°C, 1100 m^−2^ · cd illuminance and a photoperiod of 16 h per day.

### Hyperspectral time series imaging and data preprocessing

Spectral reflectance was measured with an hyperspectral imaging line scanner (spectral camera PFD V10E, Specim, Oulu, Finland) mounted on a stereo microscope foreoptic (Z6 APO, Leica, Wetzlar, Germany) with a magnification up to 7.3x (Figure 1). The line scanning spectrograph has a spectral range from 400 to 1000 nm and a spectral resolution of up to 2.73 nm. The maximum image size of the 30 μm sensor slot results in 1300 pixels per line, with a sensor pixel size of 0.0074 mm. Depending on this measurement setup and the magnification, a maximum spatial resolution of 7.5 μm per pixel was obtained. For image recording the leaf samples were placed nadir on a XY-moving stage (H105/2/0 ProScan Upright Stage, Prior Scientific, Jena, Germany), controlled with a joystick and Oasis software (Oasis Controller, Objective Imaging Ltd., Cambridge, England). The samples were illuminated by two linear light emitters (Dual line Lightlines, Schott, Mainz, Germany) with a vertical orientation of 30° and a distance of 20 cm to the sample besides the foreoptic. As a light source a 150 watt halogen tungsten lamp connected to the line lights via a non-absorbing fiber was used (DCR® Light Source EKE, Polytec, Waldbronn, Germany). Hyperspectral measurements were performed in a dark room after 60 minutes pre-heating of the light source in order to realize constant and reproducible illumination conditions. The software SpectralCube (Spectral Imaging Ltd., Oulu, Finland) was used for controlling the HSI line scanner and for acquiring the hyperspectral images. Images on the leaf surface level were taken with spectral binning 1 and spatial binning 1. Frame rate and exposure time were adjusted to the object.

The reflection in the range from 400 to 1000 nm was measured daily 3 to 14 days after inoculation (dai) with a magnification of 7.3x. For image normalization and subsequent calculation of reflectance, four hyperspectral images per sample were taken. First, a white reference bar (SphereOptics GmbH., Uhldingen-Mühlhofen, Germany) was recorded, followed the dark current image. Subsequently, the leaf sample and a corresponding dark current image were recorded. Additionally, RGB images of each leaf were taken daily with a digital camera (EOS 6D, Canon, Tokio, Japan) and a 100 mm object lens (EF Lens Ultrasonic EF 100 mm 1:2.8 L Macro IS USM, Canon, Tokio, Japan).

### Pre-processing of hyperspectral images

Because reflection data was noisy at the extremes, only data values between 420 to 830 nm were analysed. The reflectance of samples, was calculated by normalizing the images relative to the white reference reflection and the dark current measurements using the software ENVI 5.1 + IDL 8.3 (ITT Visual Information Solutions, Boulder, USA). Furthermore, the spectral signals are smoothed by employs the Savitzky-Golay filter [44]. Parameters for the smoothing process were 25 supporting points to the left and right, respectively, and a third degree polynomial. The pre-processed images were used for further analysis using ENVI 5.1 + IDL 8.3 and data mining methods.

### Spectral signature extraction, analysis and characterization

In a first step, spectral signatures of pixels from healthy and diseased regions were extracted manually. Therefore >300 pixel were extracted daily from the same area by an ellipsoid region of interest from each non-inoculated leaf. When powdery mildew pustules became visible the symptomatic area was extracted as region of interest, thus the amount of pixels extracted increased depending on symptom development.

### Data driven approach for fast analysis of hyperspectral dynamics

Following the method of Wahabzada *et al*. [36] a data driven approach was applied, allowing an automated analysis of hyperspectral data. Simplex Volume Maximization (SiVM) applied for fast and interpretable factorization [27], using an implementation based on the Python™ Matrix Factorization Module (PyMF) library (https://code.google.com/p/pymf/). SiVM represents the hyperspectral data in terms of only few extreme components determined across all images considered. Since the components are real extreme signatures, they are easily interpretable and uncover the variations existing in the data (Figure 4). The signatures within all hyperspectral images were then represented as combination of these extreme components.

Given the new representation opens door to statistical data mining on a massive scale. That is, the representation can be used to discover disease specific signatures within diseased leaves. This procedure avoids the risk of losing valuable information when selecting disease specific signatures manually at some diseased spots only. Following Wahabzada *et al*. [36] the differences for each particular signature was computed using likelihood ratios LLR(*s*) together with the distributions computed using simplex representation of the data. That is, the LLR(*s*) for a signature *s* of a diseased leaf at day *d* were computed in terms of the distributions of non-inoculated healthy leaf at day *d* and of a subsequent day *r* (we used *r = d* + 2) of the diseased leaf as the reference. For the latter days (*d ≥* 8 dai) we used the distribution determined from the image 10 dai for the diseased leaf as reference, as we assumed to observe the most diseased specific hyperspectral characteristics at this day.

For the binary maps of the location of disease spots a Gaussian filter was placed on the computed differences LLR(*s*) and set all positive values to 1 and 0 otherwise.

The leaf traces were computed by applying the Dirichlet aggregation regression on the representation determined by SiVM [45]. For interpolated mean signatures a 2-dimensional map was computed by the simplex traces approach [41]. This uncovers hyperspectral dynamics of diseased and non-inoculated (healthy) leaves of the different genotypes over time.

## Abbreviations

*Bgh*: *Blumeria graminis* f.sp. *hordei*
DAI: Days after inoculation
HSI: Hyperspectral imaging
LLR: Likelihood ratio
ML: Mildew locus
NIR: Near infrared
RH: Relative humidity
ROI: Region of interest
RSD: Relative standard deviation
SiVM: Simplex volume maximization
SWIR: Short wave infrared
VIS: Visible range
WT: Wild type
